# Egg Consumption and 4-Year Change in Cognitive Function: The Rancho Bernardo Study

**DOI:** 10.1093/geroni/igab046.3594

**Published:** 2021-12-17

**Authors:** Donna Kritz-Silverstein, Ricki Bettencourt

**Affiliations:** University of California San Diego, La Jolla, California, United States

## Abstract

The role of dietary cholesterol in cognitive decline is unclear. Eggs are a rich source of nutrients and dietary cholesterol. This study examines the association of egg consumption with 4-year change in cognitive function in 890 older, community-dwelling adults. Participants were 357 men and 533 women aged □55y (means=70.1□8.4 and 71.5□8.8, respectively, p=0.016), from the Rancho Bernardo Cohort who attended a 1988-91 clinic visit. Egg intake was obtained with a food frequency questionnaire. Cognitive function was assessed with the Mini-Mental Status Exam (MMSE), Trails B and category fluency, and reassessed in 1992-96. In this sample, rates of egg consumption ranged from never (14.0% of men, 16.5% of women) to □5/week (7.0% of men, 3.8% of women; p=0.0013). Mean 1988-91 cognitive function scores for men vs. women were 27.5 vs. 27.7 on the MMSE (p=0.08), 105.9 vs. 121.6 on Trails B (p<0.0001), and 20.2 vs. 18.2 on category fluency (p<0.0001). Sex-specific regression analyses examined associations of egg consumption with change in cognitive function. In women, after adjustment for age and education, egg intake was associated with less decline over time in category fluency (beta=-.10, p=0.01), which remained significant after adjustment for smoking, alcohol, exercise, cholesterol, calorie intake, and protein intake (p=0.02). No other associations were found in women, and no associations were observed in men before and after adjustment for covariates. Results suggest that while high in dietary cholesterol, egg consumption is not associated with decline in cognitive function. For women, there may be a small beneficial effect for verbal memory.

